# A longitudinal analysis of myopia progression and its predictors in a Ghanaian clinical cohort

**DOI:** 10.3389/fopht.2026.1878709

**Published:** 2026-07-31

**Authors:** Gifty Frimpong, Eldrick Adu Acquah, Daniel Kwao Pecku, Julian Sakyibea Yeboah, Farida Ayinbono Selase Afenyo, Augustine Ntunyin Dery, Isaiah Osei Duah Junior, Albert Kwadjo Amoah Andoh, Werner Eisenbarth, David Ben Kumah, Kwadwo Owusu Akuffo

**Affiliations:** 1 Department of Optometry and Visual Science, College of Science, Kwame Nkrumah University of Science and Technology, Kumasi, Ghana; 2 Department of Vision Science, University of Houston College of Optometry, Houston, TX, United States; 3 Department of Psychology, John R. and Kathy R. Hairston College of Health and Human Sciences, North Carolina Agricultural and Technical State University, Greensboro, NC, United States; 4 Department of Applied Science and Mechatronics, HM Hochschule München University of Applied Sciences, Munich, Germany

**Keywords:** electronic medical record (EMR), Ghana, longitudinal study, myopia, myopia - epidemiology, myopia progression, refractive error, spherical equivalence refraction

## Abstract

**Objective:**

To investigate the rate of myopia progression among a clinical cohort in Ghana.

**Methods:**

A retrospective analysis was conducted using electronic medical records (EMRs) of all patients attending a tertiary eye care center in Ghana. Annual refractive data were extracted from January 2021 to December 2023. Myopia was defined as a spherical equivalent refraction (SER) of ≤ −0.50 D in at least one eye. Myopia progression was assessed by calculating the annual change in SER between visits, with progression defined as an SER change of ≤ −0.50 D/year. Given the moderate inter-eye correlation in progression rates, both eyes were analyzed independently.

**Results:**

Of the 4,010 EMRs reviewed during the study period, 280 patients with myopia were identified. After applying the eligibility criteria, 231 patient records were included in the final analysis. The mean age of participants was 29.41 ± 14.92 years (range: 5–81 years), and the majority were female (60.6%; n = 140). The mean rate of myopia progression was −0.063 D/year in the right eye and −0.089 D/year in the left eye. Using a progression threshold of ≤ −0.50 D/year in at least one eye, 17.7% of participants were classified as progressors, while 82.3% were non-progressors. In multivariable regression analyses adjusted for sex, age remained a significant predictor of myopia progression in our cohort (B = -0.043, SE = 0.014, p = 0.001; AOR = 0.958, 95% CI: 0.933–0.983).

**Conclusion:**

In this clinical cohort, myopia progression was generally slow, with fewer than one in five patients meeting the criteria for significant progression. Older age was independently associated with a lower risk of progression, whereas sex showed no significant association. These findings indicate that myopia progression in this predominantly adult Ghanaian population is relatively modest compared with rates reported in younger populations, and identify age as a key determinant of progression in routine clinical care.

## 1 Introduction

Myopia has transitioned from a routine refractive condition to a major global public health challenge with a sustained increase in prevalence over recent decades (1). Current global projections estimate that nearly 50% of the world’s population will be myopic by 2050, with a substantial proportion at risk of developing high myopia and its associated sight-threatening complications (2); however, these estimates are derived predominantly from East Asian and Western cohorts, and their applicability to African populations remains elusive. Clinically, myopia is defined as an SER of ≤ −0.50 D (3). While the onset of myopia is clinically relevant, its longitudinal progression is of greater translational importance, as cumulative increases in refractive error are strongly associated with an elevated risk of irreversible ocular morbidity in some populations (4, 5). Myopia progression is typically characterized by a continuous shift toward more negative refractive error and is operationalized clinically as the annual change in SER. Among adults, progression risk has been linked to factors such as young adult age and greater baseline myopic severity (6), although the relative contribution of these factors appears to vary across populations (6). This trajectory reflects a multifactorial process shaped by genetic susceptibility and environmental exposures, including sustained near-work demand and reduced outdoor activity (7), though the extent to which these associations hold in African adult populations has not been well characterized.

Although the epidemiology of myopia has been extensively characterized in East Asian and European populations, evidence from Africa remains limited. East Asia has experienced an unprecedented surge in myopia prevalence, with estimates reaching up to 90% among adolescents and young adults in some urban settings (8). In Africa, by contrast, childhood myopia prevalence is consistently low, though the evidence itself is thin and methodologically skewed (9). The most comprehensive estimate derive from two related systematic reviews and meta-analyses drawing on school- and population-based studies across 19 of 54 African countries, which reported overall childhood myopia prevalence of 4.7% and high myopia of 0.6% with urban progressions reaching 17.7% by 2050 (9). Of the studies include in the estimation, only two were population-based, with the remainder relying on school-based sampling, a meaningful limitation given that sub-Saharan Africa has the highest out-of-school rate globally, and school-based designs tend to inflate estimates among those who remain enrolled, particularly at senior levels. A further systematic review highlights that non-cycloplegic refraction systematically overestimates myopia prevalence compared with cycloplegic refraction, yielding estimates of 6.4% versus 4.2%, respectively, in African school-children indicating that much of the apparent cross-country variability in African prevalence data likely reflects measurement technique rather than true biological difference (10). In Ghana specifically, school-based cross-sectional studies have reported myopia prevalence ranging from approximately 1.7% to 3.4% among children, though these figures are derived almost entirely from non-cycloplegic or mixed-method designs and therefore cannot be taken as definitive population estimates (11, 12). However, these estimates are derived largely from cross-sectional studies and therefore provide limited insight into longitudinal refractive trajectories and the determinants of progression (13). This represents a critical gap in knowledge regarding both the rate and drivers of myopic progression in African populations. The prevalence of myopia among African populace is still sparse and dominated by hospital- based samples rather than population-based sampling. In Nigeria, one of the few population-based adult studies found an overall refractive error prevalence of 33.8% among adults aged 30 and above (14), highlighting how clinic-derived data continues to dominate West African adult literature. Crucially, prospective cohort studies of adult myopia progression remain nonexistent for any sub-Saharan African population. While limited retrospective cohort data has recently emerged to provide early longitudinal baselines (15) there is still an urgent need for broader evidence from local clinical environments. To address this critical gap and expand the regional baseline, this study presents a retrospective cohort analysis of myopia progression within a Ghanaian clinical population from January 2021 to December 2023. In contrast, international data underscores the active nature of adult vision changes; for instance, a reanalysis of cross-sectional surveys in the United States estimated that adult-onset cases accounted for 17.1% of the population-level increase in myopia, with an analogous generational cohort showing at least a −1.00 D refractive shift across adulthood (16). Similarly, the Raine Study in Australia prospectively tracked a cohort from age 20 to 28, finding that 38% of young adults experienced a myopic shift of −0.50 D or greater (17). This continuous adult progression is further mirrored in European literature, such as a landmark Finnish longitudinal cohort which tracked myopes over 23 years and demonstrated that 45% of individuals exhibited persistent, ongoing myopia progression well into their twenties (18). These global findings underscore the clinical relevance of adult progression, their generalizability to African cohorts cannot be assumed, further justifying the need for localized tracking.

Characterizing progression is clinically essential, as both the severity and rate of refractive change are directly linked to the risk of vision-threatening complications, including retinal detachment, glaucoma, cataract, and myopic maculopathy (19). In the absence of longitudinal data, accurate prediction of disease burden and implementation of effective preventive strategies remain limited. This gap is particularly relevant in Ghana, where rapid urbanization, evolving educational demands, and increased near-work exposure may be reshaping refractive development patterns, although these transitions remain poorly quantified (13). From a translational standpoint, the lack of local myopia progression data constrains the development of risk stratification frameworks and delays the adaptation of evidence-based myopia control strategies. While substantial advances in pharmacological, optical, and behavioral myopia control strategies have emerged from high-prevalence regions, particularly in Asia (20), the extent to which these interventions are applicable to Ghanaian populations remains uncertain due to potential differences in baseline prevalence, progression dynamics, and environmental exposures. Establishing population-specific longitudinal evidence is therefore essential to inform clinical decision-making, guide public health planning, and support the development of context-appropriate myopia control strategies.

To this end, the present retrospective study used electronic medical records from a tertiary eye care center in Ghana to characterize myopia progression patterns in a Ghanaian clinical cohort. Specifically, the study quantified longitudinal changes in spherical equivalent refraction and examined demographic and clinical predictors of progression. Taken together, this study addresses an important gap in longitudinal evidence on myopia progression in Ghana. The findings provide insight into progression patterns within a Ghanaian clinical cohort and contribute to the emerging evidence base needed to inform future research, risk assessment, and population-specific myopia management strategies.

## 2 Materials and methods

### 2.1 Study design, population, and setting

**Figure 1:**
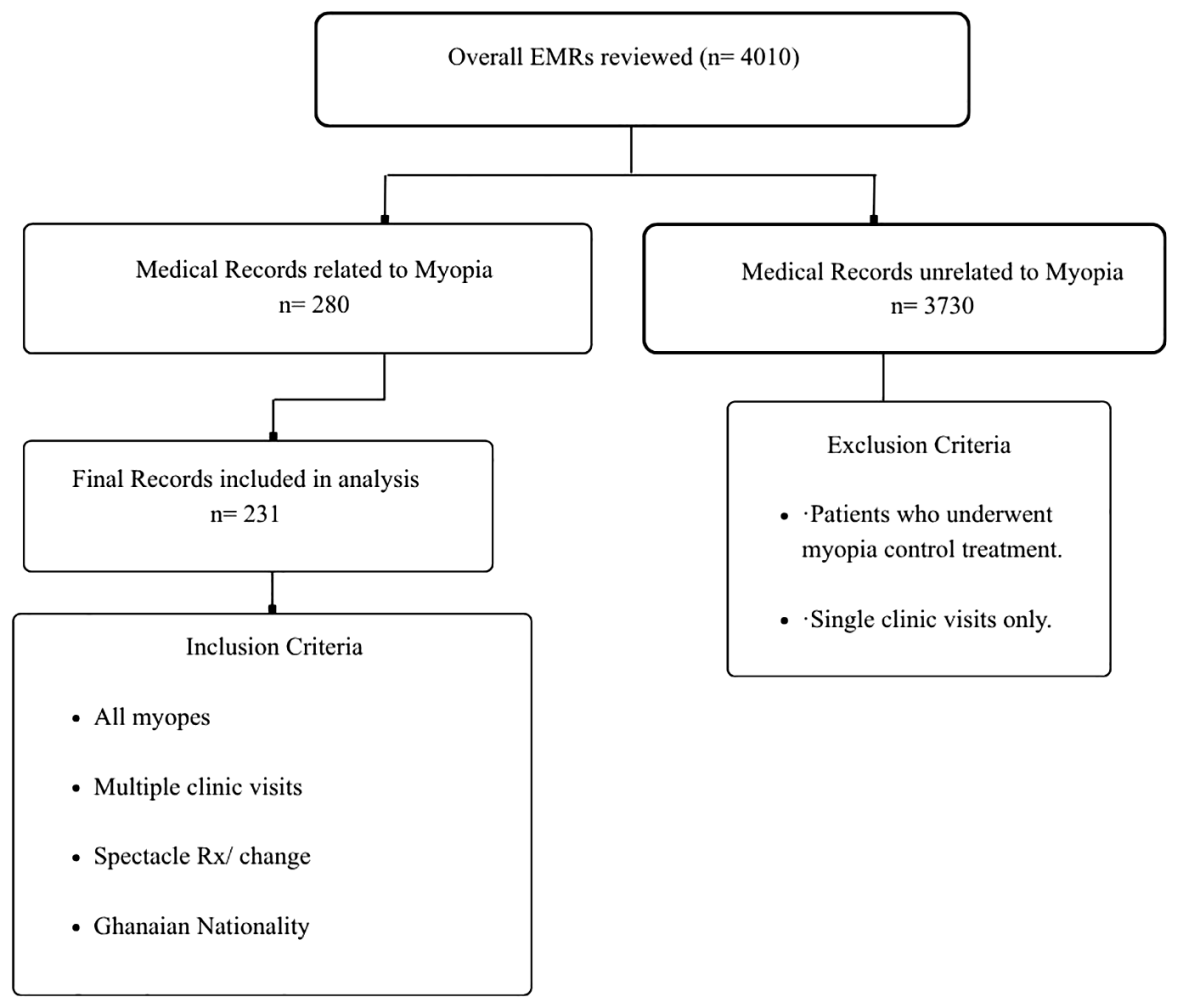
Flowchart illustrating the selection of medical records for analysis.

This retrospective longitudinal cohort study utilized EMRs from a tertiary eye care facility in Accra, Ghana. Records from patients who attended the facility between January 2021, and December 2023 were reviewed. Eligible participants were individuals with myopia who had undergone at least two eye examinations separated by a minimum follow-up period of 12 months. Myopia was defined according to the International Myopia Institute criteria as a spherical equivalent refraction (SER) of ≤ −0.50 D in at least one eye (21, 22). No age restrictions were applied for inclusion; participants of any age meeting the above criteria were eligible, resulting in a sample spanning ages 5 to 81 years. Given this wide age range, age was retained as a covariate in regression analyses to account for its association with refractive progression. Demographic and clinical data extracted from the EMRs included age at baseline, sex, occupation, number of clinic visits, unaided visual acuity, best-corrected visual acuity (BCVA), intraocular pressure (IOP), and refractive error measurements. Dilated fundus examination was performed at the discretion of the attending clinician rather than as a standardized annual protocol component; consequently, fundus findings were not uniformly available across all participants or visit intervals. Refractive error was derived from recorded sphero-cylindrical prescriptions, and SER was calculated as sphere + (cylinder/2). Visual acuity measurements recorded in Snellen notation were converted to logarithm of the minimum angle of resolution (LogMAR) units for statistical analyses. Myopia progression was quantified as the annual change in SER between visits and was defined as a change of ≤ −0.50 D/year in at least one eye, consistent with previous studies. Both eyes were retained for analysis because, although baseline refractive measurements exhibited strong inter-eye correlations, correlations for annual SER change were only moderate. Consequently, separate eye-level analyses were performed rather than selecting a single eye for inclusion (see **Figure 1**). Of note, since this was a retrospective study, sample size was determined by the available records meeting inclusion criteria rather than *a priori* calculation.

### 2.2 Measures

The primary outcome was myopia progression, defined as the change in SER (ΔSER) between baseline and follow-up visits. To account for variation in follow-up duration, the progression rate, taken as the annual change in SER (D/year), was calculated as the difference between baseline and follow-up SER divided by the number of years between baseline and follow-up visits. Mathematically, that is, progression rate = $\frac{\left(\text{Follow}-\text{up SER }-\text{baseline SER}\right)}{\text{number of years between visits}}$. The annual progression rate calculated here represents the mean rate over the entire follow-up interval and may not reflect year-to-year variation in progression. Given that not all participants were examined annually, decomposition into period-specific rates was not uniformly possible. For categorical analyses, patients were classified as either progressors or non-progressors using a threshold of -0.50 D/year annual change in SER (6, 23). Progressors were those with an annual change of ≤ -0.50 D/year in at least one eye, whereas non-progressors were those with an annual change of > -0.50 D/year.

### 2.3 Data analysis

Descriptive statistics and chi-square analysis were used to summarize demographic variables. Continuous variables were presented as means ± standard deviations, while categorical variables were expressed as frequencies and percentages. Pearson correlation coefficients were calculated to assess correlations between right and left eye measurements. Paired t-tests were used to compare baseline and follow-up SER and VA measurements. Both linear and logistic regression analyses were used to examine associations between demographic variables and myopia progression rates and status, respectively. Statistical significance was set at p < 0.05 with a 95% confidence interval.

### 2.4 Ethical consideration

The study was performed in accordance with the Declaration of Helsinki, and ethical approval for the study was obtained from the Committee on Human Research Publication and Ethics (CHRPE/AP/796/24) at the School of Medicine and Dentistry, Kwame Nkrumah University of Science and Technology. Institutional approval was obtained from the participating eye care facility. Patients’ data were anonymized prior to analysis to ensure confidentiality.

## 3 Results

### 3.1 Socio-demographic characteristics of patients

The analytical sample comprised 231 participants. The mean age was 29.41 ± 14.916 years (range: 5–81 years). Females accounted for 60.6% (n = 140), and 39.4% (n = 91) were students; all participants were of Ghanaian nationality (100%, n = 231). The mean number of hospital visits was 2.18 ± 0.416 (range: 2–4). The mean annual change in spherical equivalent refraction (SER) was −0.063 D/year in the right eye and −0.089 D/year in the left eye. Using a progression threshold of ≤ −0.50 D/year in at least one eye, 17.7% (n = 41) of participants were classified as progressors and 82.3% (n = 190) as non-progressors (see **Table 1**).

**Table 1:** Demographic distribution of patients.

Categorical variable	% (n)
Sex (n = 231)	
Male	39.4 (91)
Female	60.6 (140)
Occupational group (n = 231)	
Pensioner	1.7 (4)
Student	39.4 (91)
Public service and social workers	21.2 (49)
Business and financial sector workers	28.6 (66)
Technical and professional workers	9.1 (21)
Nationality (n = 231)	
Ghanaian	100 (231)
Progression status (n = 231)	
Progressors	17.7 (41)
Non-progressors	82.3 (190)
Continuous Variable	Mean (± SD)
Age	29.41 (± 14.916)
Number of visits	2.18 (± 0.416)
Baseline Unaided Distance LogMAR VA_OD	0.6303 (± 0.344)
Baseline Unaided Distance LogMAR VA_OS	0.5969 (± 0.366)
Baseline IOP_OD	13.782 (± 2.865)
Baseline IOP_OS	13.642 (± 2.445)
Baseline SER_OD	-2.1737 (± 1.893)
Baseline SER_OS	-2.0271 (± 1.841)
Baseline BCVA LogMAR_OD	0.0393 (± 0.094)
Baseline BCVA LogMAR_OS	0.0363 (± 0.089)
Follow-up Unaided Distance LogMAR VA_OD	0.6257 (± 0.345)
Follow-up Unaided Distance LogMAR VA_OS	0.6024 (± 0.375)
Follow-up IOP_OD	13.68 (± 2.851)
Follow-up IOP_OS	13.672 (± 2.634)
Follow-up SER_OD	-2.2798 (± 2.005)
Follow-up SER_OS	-2.1434 (± 2.031)
Follow-up BCVA LogMAR_OD	0.0375 (± 0.127)
Follow-up BCVA LogMAR_OS	0.021 (± 0.065)
Annual Change in SER_OD	-0.063 (± 0.390)
Annual Change in SER_OS	-0.0887 (± 0.575)

n, frequency; %, percentage frequency; SD, standard deviation; LogMAR, logarithm of the minimum angle of resolution; OD, Oculus dexter (right eye).

OS, oculus sinister (left eye); VA, visual acuity; SER, spherical equivalent refractive error; IOP, intraocular pressure.

### 3.2 Correlation between eyes

Inter-eye correlations were assessed for baseline and follow-up ocular parameters. At baseline, significant inter-eye correlations were observed for unaided distance visual acuity (r = 0.777, p < 0.001), intraocular pressure (IOP; r = 0.759, p < 0.001), spherical equivalent refraction (SER; r = 0.865, p < 0.001), and best-corrected visual acuity (BCVA; r = 0.675, p < 0.001). At follow-up, significant inter-eye correlations were observed for unaided distance visual acuity (r = 0.777, p < 0.001), IOP (r = 0.879, p < 0.001), and SER (r = 0.819, p < 0.001). Inter-eye correlations were lower for follow-up BCVA (r = 0.440, p < 0.001) and for annual change in SER (r = 0.424, p < 0.001) see **Table 2**.

**Table 2:** Correlations between eyes for key measures and outcome variable.

Variables	Inter-eye correlations	
	r	p-value
Baseline Unaided Distance LogMAR VA	0.777	<0.001
Baseline IOP	0.759	<0.001
Baseline SER	0.865	<0.001
Baseline BCVA LogMAR	0.675	<0.001
Follow-up Unaided Distance LogMAR VA	0.777	<0.001
Follow-up IOP	0.879	<0.001
Follow-up SER	0.819	<0.001
Follow-up BCVA LogMAR	0.44	<0.001
Annual Change in SER	0.424	<0.001

LogMAR, logarithm of the minimum angle of resolution; OD, Oculus dexter (right eye); OS, oculus sinister (left eye). VA, visual acuity; SER, spherical equivalent refraction; IOP, intraocular pressure.

### 3.3 Spherical equivalent refraction changes

**Figure 2:**
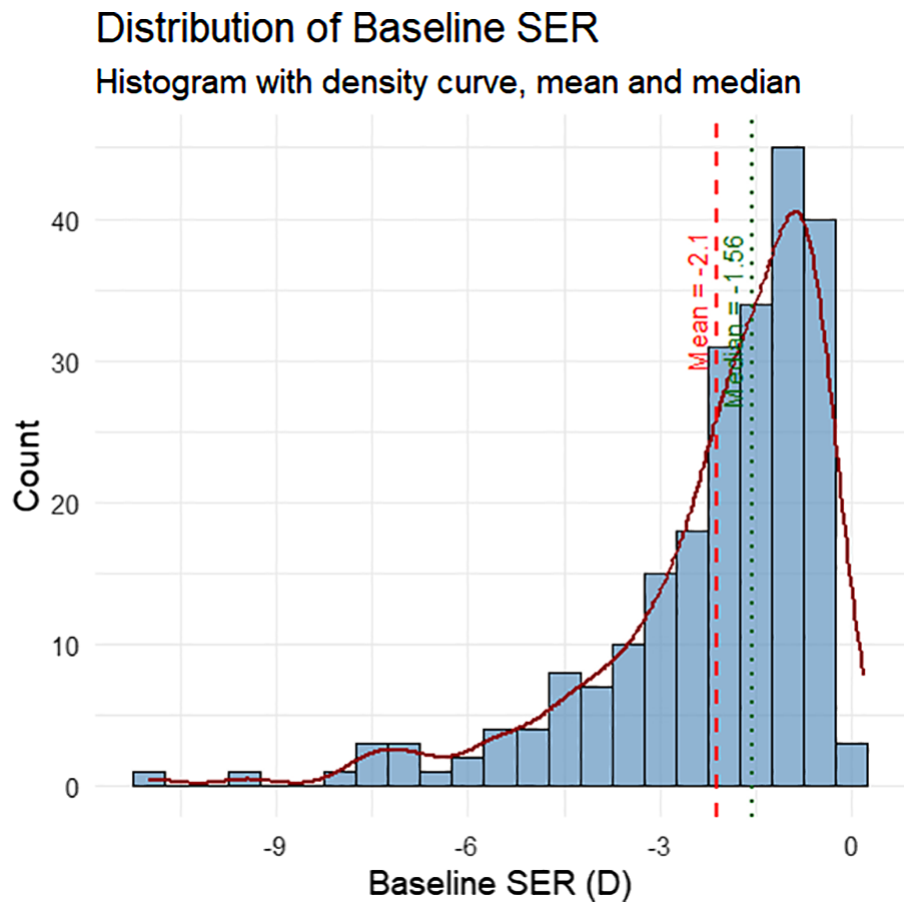
Histogram illustrating the baseline spherical equivalent refraction (SER) distribution.

Given the moderate inter-eye correlation in annual change in SER, analyses were performed separately for each eye. The mean baseline and follow-up SER were −2.17 ± 1.893 D and −2.28 ± 2.005 D for the right eye, and −2.03 ± 1.841 D and −2.14 ± 2.031 D for the left eye, respectively. Baseline and follow-up SER were strongly correlated in both eyes (OD: r = 0.974, p < 0.001; OS: r = 0.884, p < 0.001). Over the study period, mean SER change was −0.11 ± 0.457 D in the right eye (t = 3.524, p = 0.001) and −0.12 ± 0.952 D in the left eye (t = 1.858, p = 0.065). The baseline SER distribution ranged primarily from −3.0 D to 0.0 D, with a mean of −2.1 D and a median of −1.56 D (see **Figure 2**). The distribution was unimodal, with most observations concentrated in the mild-to-moderate myopia range, and relatively fewer cases in the high myopia range (−6.0 D to −9.0 D).

### 3.4 Unaided visual acuity and best-corrected visual acuity changes

The mean baseline and follow-up unaided VA in the right eye were 0.630 ± 0.344 and 0.626 ± 0.345 logMAR, respectively, while corresponding values for the left eye were 0.597 ± 0.366 and 0.602 ± 0.375 logMAR. Baseline and follow-up unaided VA were strongly correlated in both eyes (OD: r = 0.846, p < 0.001; OS: r = 0.866, p < 0.001). The mean change in unaided VA was 0.005 ± 0.191 logMAR in the right eye and −0.005 ± 0.192 logMAR in the left eye. Neither change reached statistical significance (OD: t = 0.314, p = 0.753; OS: t = −0.471, p = 0.638). The mean baseline and follow-up BCVA in the right eye were 0.039 ± 0.094 and 0.030 ± 0.086 logMAR, respectively, while corresponding values for the left eye were 0.034 ± 0.084 and 0.021 ± 0.065 logMAR. Baseline and follow-up BCVA were correlated in both eyes (OD: r = 0.726, p < 0.001; OS: r = 0.599, p < 0.001). The mean change in BCVA was 0.009 ± 0.067 logMAR in the right eye and 0.013 ± 0.069 logMAR in the left eye. Both changes were statistically significant (OD: t = 2.049, p = 0.042; OS: t = 2.934, p = 0.004).

### 3.5 Predictors of myopia progression

**Figure 3:**
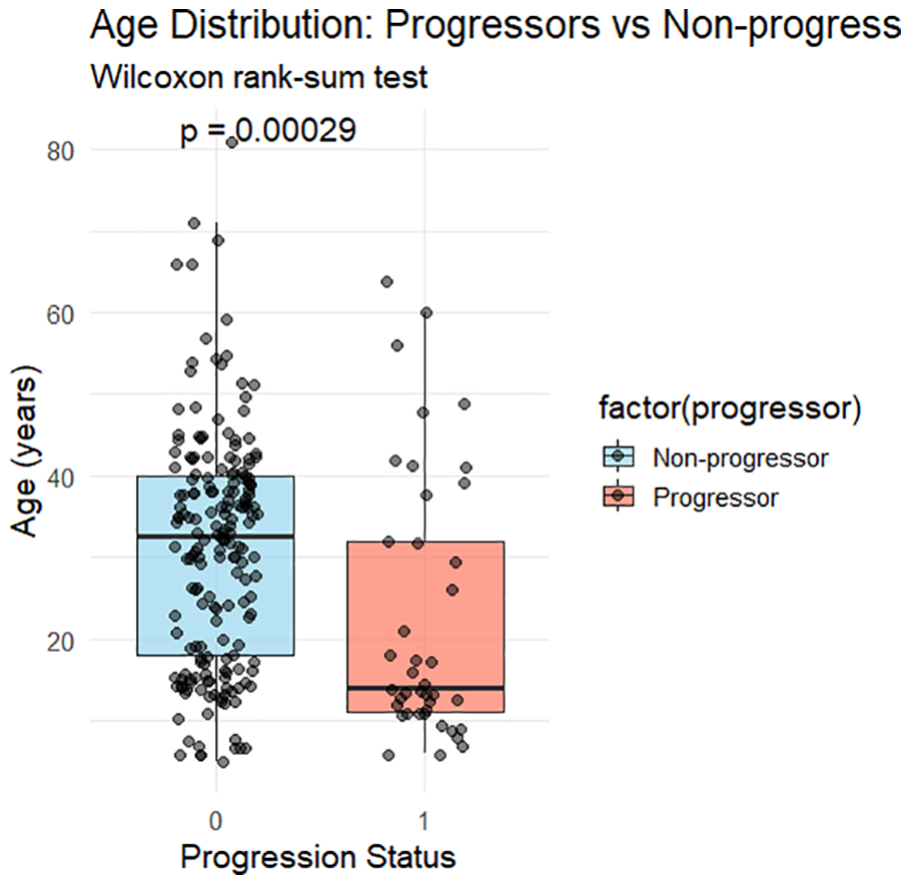
Box plot showing the age distribution of progressors versus non-progressors.

**Figure 4:**
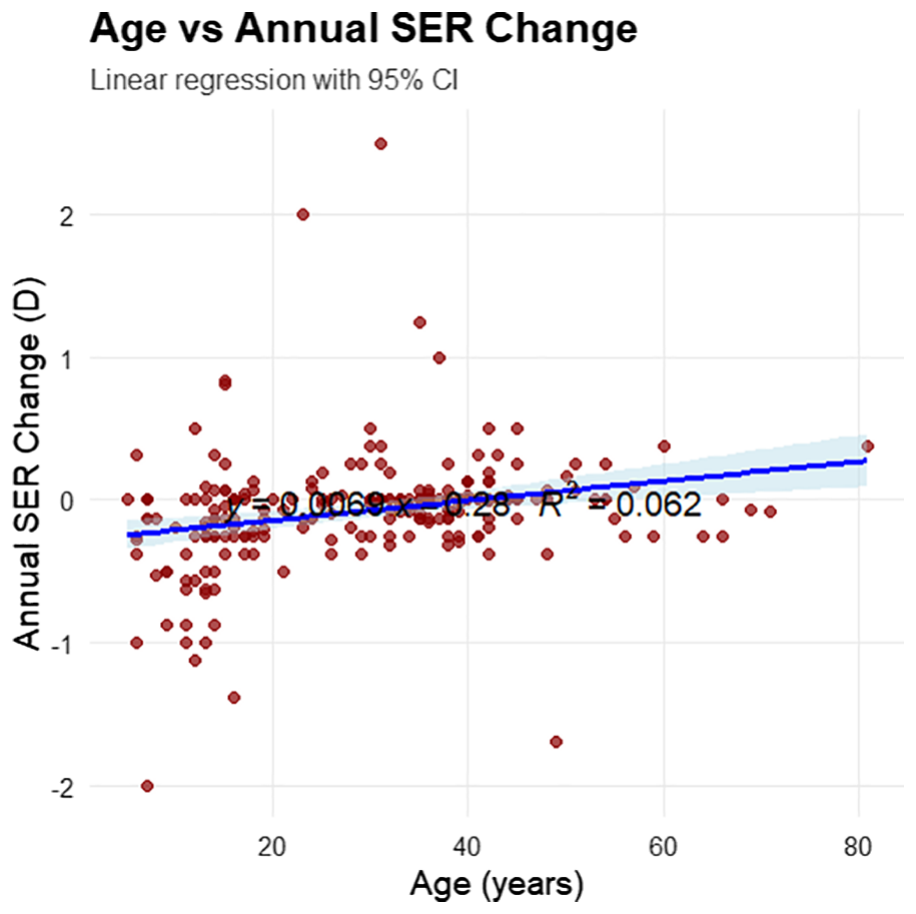
Scatterplot illustrating the association between age and annual SER change.

Factors associated with myopia progression were evaluated using linear regression for annual SER change and logistic regression for progression status. Baseline SER was included as a covariate in all models. Generalized estimating equations with an exchangeable correlation structure and robust standard errors were used to account for inter-eye correlation. Age was associated with annual SER change (B = 0.007, S.E = 0.002, Wald χ² = 19.29, p < 0.001), whereas sex and baseline SER were not associated with annual SER change. The estimated inter-eye correlation was 0.367 (see **Tables 3**, **4**). Progressors had a lower median age than non-progressors (14.0 years vs. 32.5 years, p < 0.001; Wilcoxon rank-sum test; **Figure 3**). In univariate logistic regression analyses, age was associated with progression status (B = −0.043, S.E = 0.013, p = 0.002), with increasing age associated with lower odds of progression (COR = 0.958, 95% CI: 0.933–0.984). Baseline SER in the right eye (B = −0.275, SE = 0.086, p = 0.001; COR = 0.760, 95% CI: 0.641–0.900) and left eye (B = −0.199, S.E = 0.085, p = 0.019; COR = 0.820, 95% CI: 0.694–0.967) were also associated with progression status. Sex was not associated with progression status (p = 0.765). In the multivariable model including age, sex, and baseline SER, age (B = −0.048, S.E = 0.015, p = 0.001; AOR = 0.953, 95% CI: 0.926–0.981) and baseline SER in the right eye (B = −0.560, SE = 0.190, p = 0.003; AOR = 0.571, 95% CI: 0.393–0.829) remained associated with progression status. Sex (p = 0.610) and baseline SER in the left eye (p = 0.065) were not associated with progression status in the adjusted model (see **Table 5**). Further, **Figure 4** shows the relationship between age and annual SER change (β = 0.0069, R² = 0.062).

**Table 3:** Predictors of myopia progression rate .

Parameter	B	S.E.	95% CI	p-value
Age	0.007	0.0017	0.004-0.001	<0.001
Sex				
Male	Ref			
Female	-0.08	0.0531	-0.208	0.133
Baseline_SER	-0.008	0.0207	-0.081	0.705

Generalized estimating equations was used to estimate myopia progression; SE, standard error of the mean. SER, spherical equivalent refractive error; 95% CI, ninety-five percent confidence interval.

**Table 4:** Inter-eye correlation using generalized estimating equations.

Variable	Inter-eye correlation	
Eye	OD	OS
OD	1.000	0.367
OS	0.367	1.000

OD, Oculus dexter; OS, Oculus sinister.

**Table 5:** Predictors of myopia progression status using logistic regression analysis.

Variable	Univariate logistic regression					Multivariate logistic regression				
Model statistics	B	S.E	COR	95% CI	p-value	B	S.E	AOR	95% CI	p-value
Age	-0.043	0.013	0.958	0.933 – 0.984	0.002	-0.048	0.015	0.953	0.926 – 0.981	0.001
Sex										
Male	Ref									
Female	-0.105	0.35	0.901	0.453 – 1.789	0.765	-0.191	0.374	0.826	0.397 – 1.720	0.61
Baseline SER_OD	-0.275	0.086	0.76	0.641-0.900	0.001	-0.56	0.19	0.571	0.393-0.829	0.003
Baseline SER_OS	-0.199	0.085	0.82	0.694-0.967	0.019	0.347	0.188	1.415	0.978-2.046	0.065

S.E, standard error of the mean; COR, crude odds ratio; AOR, adjusted odds ratio; CI, confidence interval.

## 4 Discussion

This study examined myopia progression in a Ghanaian clinical cohort using longitudinal electronic medical records. By assessing SER, unaided visual acuity, and BCVA, we provide longitudinal data on refractive change in a Ghanaian clinical population, addressing a gap in evidence where most regional studies have focused on prevalence rather than progression. The mean annual SER change was −0.063 D/year in the right eye and −0.089 D/year in the left eye, with 82.3% of participants classified as non-progressors using a threshold of ≤ −0.50 D/year. This threshold is consistent with commonly used definitions of clinically meaningful myopia progression in epidemiological and clinical studies (6, 23), although its application across a wide age range may introduce classification heterogeneity. Overall, the observed rates indicate low-magnitude refractive change in this cohort.

A previous Ghanaian retrospective cohort study by Kyei and colleagues reported a higher proportion of progression (56.8%) over a four-year follow-up period, with younger age identified as a key determinant of progression and variation across ethnic groups (15). Differences between that study and the present analysis likely reflect variation in age structure and clinical composition, with younger cohorts typically exhibiting faster refractive change (15). In contrast, the present cohort, with a mean age of 29.4 years, demonstrated lower progression rates and a predominance of refractive stability. Together, these findings support age as a central determinant of refractive change and are consistent with the broader literature describing deceleration of myopia progression with increasing age.

Comparisons with international data further contextualize these findings (24). For instance, whiles a study in the United States have reported higher rates of adult myopia progression (approximately −0.10 to −0.25 D/year depending on subgroup) (24), the present analysis from the Ghanaian cohort exhibited smaller annual changes. Despite differences in magnitude, both datasets indicate that adult progression is generally limited, with clinically meaningful change occurring in a minority of individuals. The proportion classified as progressors in this study (17.7%) was lower than in some reports, although this may be influenced by the stricter progression threshold applied and the older age distribution of the sample. Across studies, age consistently emerges as an inverse predictor of progression risk.

In comparison with other pediatric and adolescent population studies elsewhere, the progression rates observed here are substantially lower. Previous studies have reported annual myopic shifts ranging from approximately −0.40 D/year to −0.67 D/year in younger cohorts (25, 26), with higher rates frequently reported in East Asian populations (27, 28). The lower rates observed in the present study are consistent with age-related slowing of refractive change, as myopia progression typically stabilizes in early adulthood (24, 29, 30).

A small but statistically significant myopic shift was observed in the right eye over the follow-up period (−0.11 D), while the left eye showed a non-significant change (−0.12 D). Given that follow-up duration ranged from 2 to 4 years, these changes likely reflect minimal cohort-level refractive drift rather than sustained asymmetric biological progression. The similarity in magnitude between eyes further suggests that observed differences are unlikely to be clinically meaningful. These findings are supported by logistic regression results, which demonstrated a significant inverse association between age and progression risk.

Inter-eye correlations were strong for baseline and follow-up measures of SER, intraocular pressure, and unaided visual acuity, consistent with known symmetry in ocular development (31). The moderate correlation observed for annual SER change (r = 0.424) supports the analytic decision to evaluate eyes separately and suggests that short-term refractive change may be influenced by eye-specific variability or measurement noise. Overall, the high concordance between eyes for structural and functional parameters reinforces the reliability of monocular measures in epidemiological analyses.

Unaided visual acuity remained stable over time in both eyes, with no statistically significant change observed. This is consistent with prior studies showing that small refractive shifts do not necessarily translate into measurable changes in unaided vision in mild-to-moderate myopia (5). In contrast, BCVA showed small but statistically significant changes in both eyes. The magnitude of change (0.009–0.013 logMAR) is below established test–retest variability thresholds for logMAR acuity (approximately 0.05–0.10 logMAR) (32), suggesting limited clinical relevance. These changes may reflect measurement variability, differences in refractive correction at follow-up, or testing conditions rather than true changes in visual function. Previous literature similarly indicates that BCVA is generally preserved in the absence of ocular pathology, even in the presence of refractive change (33).

In this study, age was the only consistent predictor of myopia progression across regression models. Increasing age was associated with less negative annual SER change and lower odds of progression, corresponding to an approximate 4% reduction in progression risk per additional year of age. These findings are consistent with established evidence that myopia progression is most pronounced in childhood and decreases with age (5, 24, 30). Sex was not associated with progression in either univariate or multivariable models, aligning with prior studies reporting minimal sex-related differences in refractive development (34, 35). Together, these suggest myopia progression in this predominantly adult Ghanaian cohort is limited in magnitude, with age acting as the primary determinant of progression risk.

### 4.1 Study strengths and limitations

This study leverages longitudinal clinical data to characterize myopia progression in a Ghanaian cohort, providing evidence from a population in which such data remain limited. The use of routine clinical records offers practical insight into refractive change over time in a Ghanaian setting. However, some limitations should be considered. The retrospective design limited control over measurement conditions, including the absence of cycloplegic refraction, axial length data, and standardized ocular imaging, and routine fundus evaluation, which was performed at the clinician’s discretion rather than at fixed intervals. The lack of cycloplegic refraction is particularly important, as manifest refraction is influenced by accommodation and may differ from cycloplegic values by 0.25–0.75 D, especially in younger individuals (36). Consequently, some observed changes (−0.063 to −0.089 D/year) may reflect measurement variability rather than true axial progression. Although the cohort spanned a wide age range (5–81 years), regression analyses indicated that progression was concentrated among younger participants, with age associated with both smaller annual SER change and lower odds of progression, a pattern inconsistent with major confounding from age-related lenticular changes. Nonetheless, in the absence of cataract grading or axial length data, a residual contribution from lens-related refractive shift in the subset of older participants who meet the progression threshold cannot be fully excluded and should be considered in the interpretation of findings. Systemic diseases such as diabetes mellitus, which may transiently alter refractive error, were also not systematically documented in the EMR system and could not be controlled for, and their potential influence on the observed progression rates cannot be excluded.

The moderate inter-eye correlation for annual SER change (r = 0.424) led to separate eye analyses; however, this does not fully account for within-subject clustering. No exclusion criteria were applied for astigmatism, which may have influenced SER-derived estimates of progression. Follow-up duration varied across participants, and the single-center design may limit generalizability. The absence of axial length and behavioral data (e.g., near work, outdoor exposure) further constrains mechanistic interpretation.

### 4.2 Implications for practice and future

From a clinical perspective, the low progression rates observed suggest that most adults in this setting may not require myopia control interventions, but continued monitoring remains important, particularly for younger patients and higher myopes who are at higher risk of progression. Future research should focus on prospective and multicenter longitudinal studies that incorporate standardized cycloplegic refraction, axial length measurements, and detailed environmental exposure data. Such studies will be critical for fully characterizing patterns of myopia progression in African populations and informing targeted prevention and management strategies.

## 5 Conclusion

In this Ghanaian clinical cohort, myopia progression was generally low in magnitude, with the majority of participants demonstrating refractive stability over the study period. Age emerged as the primary determinant of progression, with younger individuals showing greater susceptibility to refractive change, while sex was not associated with progression outcomes. Overall, the findings indicate that clinically significant myopia progression is relatively uncommon in this predominantly adult population. These results provide baseline longitudinal evidence from a Ghanaian setting and underscore the need for prospective studies incorporating cycloplegic refraction, axial length measurements, and environmental exposures to more precisely characterize myopia progression in African populations. Beyond its local relevance, this study contributes to the global evidence base by extending longitudinal myopia data to underrepresented populations, thereby improving the generalizability of refractive epidemiology and informing more inclusive models of myopia progression.

## Data Availability

The dataset that supports the conclusion of this paper are available from the corresponding authors upon reasonable request.
